# An Experimental Parametric Optimisation for Laser Engraving and Texturing to Integrate Zirconia Ceramic Blocks into Stainless Steel Cutlery: A State-of-the-Art Aesthetically Improved Perspective

**DOI:** 10.3390/ma17102452

**Published:** 2024-05-19

**Authors:** Vipin Richhariya, Georgina Miranda, Filipe Samuel Silva

**Affiliations:** 1 Center for MicroElectroMechanical Systems (CMEMS-UMinho), Campus de Azurém, University of Minho, 4800-058 Guimarães, Portugal; fsamuel@dem.uminho.pt; 2 CICECO, Aveiro Institute of Materials, Department of Materials and Ceramic Engineering, University of Aveiro, 3810-193 Aveiro, Portugal; gmiranda@ua.pt

**Keywords:** steel, zirconia, green compact, cutlery, Laser Surface Texturing (LST), engraving, pattern, roughness, material removal, fluence

## Abstract

Cutlery and flatware designs are an everchanging phenomenon of the manufacturing industry. Worldwide hospitality businesses demand perpetual evolution in terms of aesthetics, designs, patterns, colours, and materials due to customers’ demands, modernisation, and fierce competition. To thrive in this competitive market, modern fabrication techniques must be flexible, adoptive, fast, and cost effective. For decades, static designs and trademark patterns were achieved through moulds, limiting production to a single cutlery type per mould. However, with the advent of laser engraving and design systems, the whole business of cutlery production has been revolutionised. This study explores the possibility of creating diverse designs for stainless steel 304 flatware sets without changing the entire production process. The research analyses three key laser process parameters, power, scanning speed, and number of passes, and their impacts on the resulting geometry, depth of cut, surface roughness, and material removed. These parameters are comprehensively studied and analysed for steel and zirconia ceramic. The study details the effects of power, scanning speed, number of passages, and fluence on engraved geometry. Fluence (power*number of passages/scanning speed) positively influences outputs and presents a positive trend. Medium power settings and higher scanning speeds with the maximum number of passages produce high-quality, low-roughness optimised cavities with the ideal geometric accuracy for both materials.

## 1. Introduction

Cutlery has proved to be a simple but extremely useful tool for generations around the world to serve, cook, or consume edibles [1,2]. In terms of materials, stainless steel has become a preferred metal for most cutlery, as it is easy to maintain, non-reactive, and sturdy [2,3]. Stainless steel-304 (SS-304) is widely used in fields like food processing, automobile parts, aerospace, and nuclear sectors due to its excellent mechanical properties and distinguished corrosion resistance behaviour [4,5,6,7,8]. In the cutlery industry, aesthetic is a major factor considered for the selection and purchase of items, thus, we see a huge variety of cutlery available in different shapes, sizes, designs, colours and metals [2,9].

Globally, researchers and industrialists are on a constant quest to improve the lifespan of metallic objects, tools, and housewares using surface engineering, as well as promoting better aesthetics, patterns, and colours of these components [10,11,12]. Various techniques have been used to modify the surface of steel components, namely: laser ablation, Electro-Chemical Machining (ECM), Electrode Discharge Machining (EDM), sandblasting, chemical etching, surface coatings, and plasma [13,14]. Lasers (Carbon Dioxide (CO2), Yttrium Aluminium Garnet (YAG), Fibre) in the form of machining, texturing, and treatment came as a breakthrough to alter these surfaces in additive and subtractive ways. Laser is one way to obtain surface characteristics like wettability, roughness, and engraved geometries without altering the bulk properties of the substance [15]. Lasers are also independent of the hardness of the working materials and can easily process from metal to ceramics to polymers without much difficulty. The application of lasers can significantly reduce the burden on the production lines that need to be changed entirely due to the addition of or reduction in a few steps along the process. However, the use of laser is still amateur when it comes to houseware industries, specifically the designing/patterning of aesthetically appealing silverwares. Decades-old moulding processes to produce permanent designs in bulk can be improved drastically by introducing lasers into these industries.

Zirconia (ZrO_2_), on the other hand, has always been the centre of attention in medical fraternity because of its antibacterial properties and inert nature [16,17,18,19]. Nowadays, the use of zirconia as a coating element or base material in prosthesis and dental implants is quite common [20,21,22,23,24,25]. Green zirconia compacts are highly machinable through lasers, less time-consuming, and offer extraordinary detailing and ease. These favourable characteristics and properties can be exploited not only in the biomedical and implant industries, but possibly also in the hospitality market, especially for flatware. Sintered zirconia is extremely hard, has rigorous wear and impact resistance, and is antimicrobial. The designs, patterns, shapes, looks, colours, and materials of cutlery have changed extraordinarily in recent times by virtue of customer demand and aesthetic taste. Different cookery shows, celebrity chefs, food vlogs, and media circles have created a market for sophisticated housewares and a parallel economy has risen to existence in the name of food world.

At the different usages of zirconia incorporated with SS-304 using laser can offer a transformation in the aesthetics and designs of flatware. For this reason, it is vital to study the process and interactions of laser with both the substances in detail. Performing precision milling to machine sintered zirconia has seen a trend recently, however, high-speed milling produces very high temperatures and milling tools exhaust quickly [26]. Other precision-impaired machining processes such as griding, turning, and spark plasma are also used, depending on the application [27]. EDM is efficient, precise, and heat dissipative, but unfavourable for zirconia due to its lack of electric conductivity. Spark plasma is used to impart conductivity to zirconia and then worked up by EDM. These merging processes are generally expensive and complicated in nature [28]. Another mode for machining zirconia is not to remove material by machining, but instead by 3D printing using Digital Light Processing (DLP) [29]. Based on the same DLP-Stereolithographic and thermoplastic 3D printing, zirconia ceramic parts with intricate shapes can be manufactured, however, optimisation complexity from slurry preparation to sintering needs to be perfected [30,31].

Laser engraving and cutting processes present a resolve to machine zirconia and SS-304 economically, intricately, with a very high production rate, and accurately and precisely. A deeper understanding of laser parameters and their effects must be described to operate with SS-304 and ZrO_2_. Zirconia can be machined in a green state followed by sintering in a furnace to produce the desired shapes and sizes [32]. Pulsed lasers are a good choice for highly temperature-reacting and oxidising substances, because materials like zirconia or alumina start sintering with a continuous laser beam or oxidise and spoil the surface finish of steels [33]. Utilising a fibre laser to engrave and texture is common due to their high fluence, ease in metal absorbance wavelength, and cutting capacity. Studying the relationships among power, scanning speed, number of passes, frequency, and other relevant laser parameters is vital to attain the desired dimension and accuracy [34,35].

This study aims to use single Nd:YV04 Fibre Laser engraving equipment to engrave SS-304 cutlery and remove zirconia blocks from a green compact, followed by sintering. These different coloured zirconia blocks are to be cut in larger dimensions in order to attain the required size after sintering. The objective of this study is to evaluate the influence of laser parameters (power, scanning speed, and number of passes or loops) on the dimensional accuracy, i.e., geometry, depth obtained, volume removed or Material Removal (MR), and surface roughness of SS-304 and zirconia. The grooves created on SS-304 will accommodate zirconia blocks in different designs, colours, shapes, patterns, and locations. Though the zirconia blocks were obtained by green compact cutting, we analysed the cavity of the zirconia for its roughness, dimensions, and geometry. This was performed for ease of analysis, because this groove will have approximately similar surface characteristics, depth, geometry, and area after removal of the blocks, and it is very hard to study 2 × 2 mm miniature blocks [36,37,38,39]. These design patterns were inspired by Portuguese sidewalk designs (*Calçada Portuguesa: The art of Portuguese pavement*). A full factorial Design of Experiments (DOE) was used to sort out the number of experiments and different combinations of the parameters, which were further analysed based on the output variables. The experiments were conducted simultaneously for SS-304 and zirconia all throughout the study.

This work is a state-of-the-art experimental study to replicate design patterns on silverware by introducing zirconia blocks. The uniqueness of the study lies in engraving the stainless-steel cutlery with intricate and aesthetic patterns inspired by the special characteristics of a region, place, or a country. Embroidered patterns and designs have always inspired designers, jewellers, architects, engineers, and scientists to adopt and adapt symbolic representations of specific times and places. The use of laser engraving and texturing tools for producing such effects is a novel approach due to performing subtractive and additive manufacturing at the same time for two different natured substances. Furthermore, parametric optimisation for a metal and a ceramic together by comparing their interactions with laser irradiations is an added advantage of this study. The study might prove to be incredibly interesting for industries to engrave and patternlike collection coins, ceramic tiles, and metal surface designs with the required roughness, quality, dimensions, and geometries, without hampering the surface by oxidation or bulk melting. We hope this study helps and complements the manufacturing community with optimisation and patterning and provides a different perspective on aesthetics in day-to-day life.

## 2. Materials and Methods

In this work, alumina-toughened zirconia graded as ATZ 20/80 2.5YSZ BA from NANOE Ceramics, Ballainvilliers, France was used. The characteristics of the zirconia are mentioned in Table 1. Green zirconia palettes were prepared by powder metallurgy (PM) cold pressing. Difficulties lying in processing the sintered zirconia were an immense challenge and the processing of the green zirconia was conducted by laser. Nevertheless, the zirconia was sintered during laser treatment. Zirconia powder was placed inside a hardened steel cylindrical die-punch system with a 40 mm diameter and compressed uniaxially at a 200 MPa pressure for 60 s to obtain a 5 mm thickness. After the compaction of the palettes, the pressure was gradually released and used for further laser-based processes. The composition of the SS-304 (cutlery material) cut into 40 × 40 × 4 mm is depicted in Table 2.

**Figure 1:**
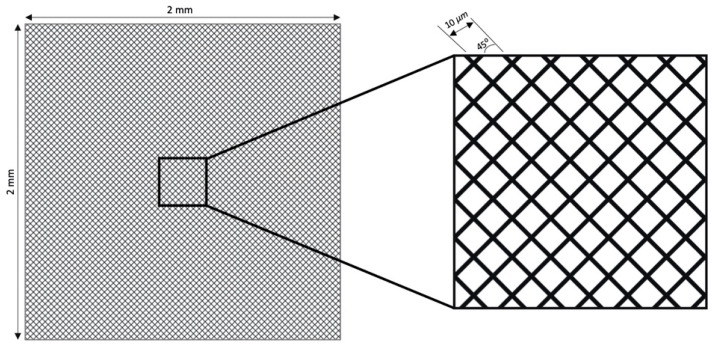
Schematic of 45° inclined cross-hatched micro-pattern used for the material removal.

**Figure 2:**
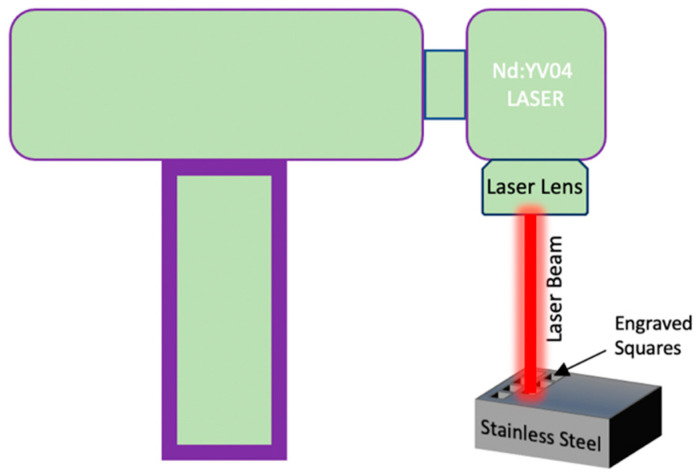
Schematic representation of laser engraving on SS-304. Laser engraving will be performed on zirconia palettes removing stainless steel and replacing it with zirconia.

Laser Surface Texturing (LST) was executed using an Nd: YV04 (Model: XM-30D Fiber Laser Marking Machine, Wuhan, China) with a maximum power of 30 watts, spot size of 10 μm, pulse width of 10 μs, and 1064 nm wavelength. The textures were produced in the form of square cavities of 2 × 2 mm. These grooves were produced by equally spaced 10 μm cross-hatched lines inclined at 45° on EzCAD laser software (JCZ, Beijing, China), as shown in the Figure 1. A schematic depicts the laser setup in Figure 2, which has a focal distance of 160 mm. To remove the disintegrated material from the textured surface and avoid the oxidation or reaction of the substrate, an argon inert atmosphere was maintained during the whole LST. As the minimum spot diameter/spot width of the laser was 10 μm, the cut line width was a minimum 10 μm. The parameters considered were power, scanning speed, and number of passes, keeping the frequency at 20 kHz. As the zirconia was in the green state, it was advocated not to use laser wobbles, as wobbles would eventually increase the area of the removed materials with greater-intensity sintering of the periphery and a Heat-Affected Zone (HAZ) produced. For zirconia, laser power levels of 6, 9, and 12 watts were used alongside scanning speeds of 500, 1000, and 1500 mm/s with varying numbers of passes of 50, 100, and 150. However, for the harder SS-304, higher power levels of 15, 22.5, and 30 watts and greater numbers of loops of 400, 800, 1200 were used by keeping the scanning speeds same. The number of runs for the experiments was designed by DOE’s full factorial design for 3 factors at 3 levels (3^3^ = 27). Table 3 and Table 4 show 27 combinations of experiments or run orders for the zirconia and steel, respectively. After texturing, to observe the cavities, the cross-sections of the specimens were polished with SiC abrasive papers from 1200 to 4000 mesh and ultrasonically cleaned (only SS-304) in an Iso-Propyl Alcohol (IPA) bath for 5 min before performing the surface analysis. Laser fluence was also calculated to figure out the combined effect of input variables based on Equation (1).

(1) $$Laser\;Energy\;Fluence\;(F)=\frac{Power\;(watts)}{Scanning\;Speed\;(mm/s)\times Interspacing\;(mm)}\times Loops$$

where Fluence (F, J/mm^2^), Power (P, watts), Scanning Speed (S, mm/s), and Interspacing (I, mm) were between the adjacent lines. To perform this study, the following equipment were used: (i) cold press, (ii) fibre laser engraving machine, (iii) ultrasonic vibrator, and (iv) rotary polishing machine.

## 3. Results and Discussions

A highly productive process is one that results in more material removal with a good cut quality in less time. Parameters like surface roughness and geometrical error are significant, because higher roughness values of the removed zirconia blocks will help to create interlocking with the SS-304 grooves’ roughness and adhesive used. Nonetheless, a very high roughness of the SS-304 groove wall or zirconia block surface can also pose problems during block accommodation. Simultaneously, geometry and errors in geometry (difference between achieved and ideally drawn dimensions) are crucial because of dimensional accuracy. With optimised laser parameters, we can achieve a balance between the quality and MR of the groove/block.

All 27 grooves were produced on the single palette by laser engraving, keeping a sufficient distance between them so as not to overlap the HAZs and geometries. The input parameters were power in watts, scanning speed in mm/s, and number of passes, maintaining the wobble diameter, wobble amplitude, and frequency of the laser constant. The output parameters were the average surface roughness in microns, average depth in mm, MR to obtain productivity in mm^3^, and periphery as the geometry of the grooves in mm. Three-dimensional optical profilometry was used for the parametric analyses and to obtain values for the output. All values were taken thrice and averaged to enhance the precision of the calculations. The HAZ became irrelevant to study for this application because of the controlled inert atmosphere and the use of zirconia ceramic [33]. Regression curve fittings for obtaining the best fit regression lines were performed on OriginProLab (OriginLab, Northampton, MA, USA) and Minitab for DOEs.

### 3.1. Findings for Zirconia

**Figure 3:**
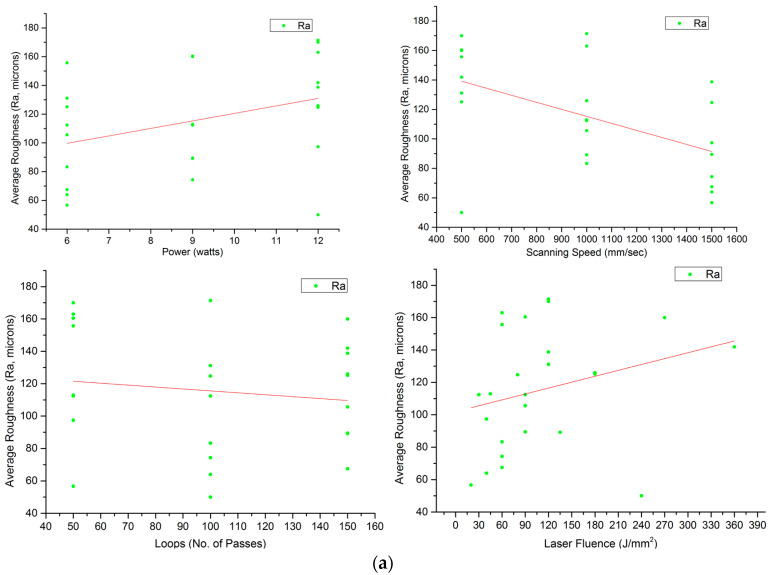

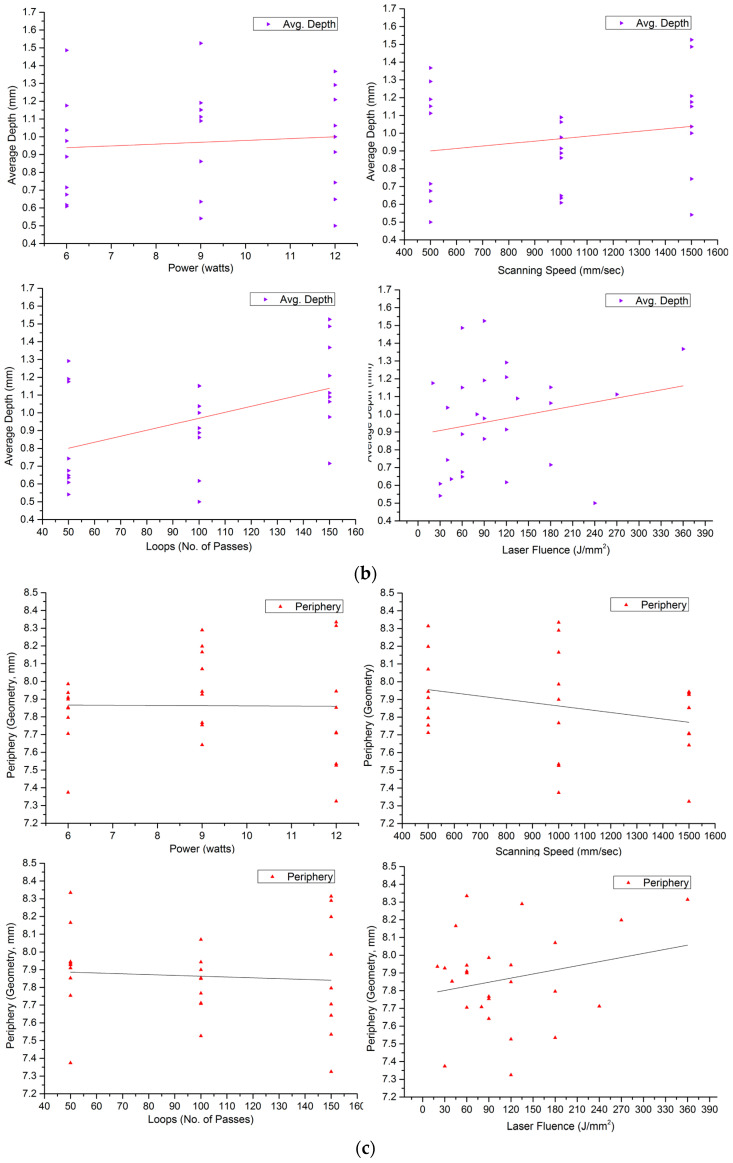

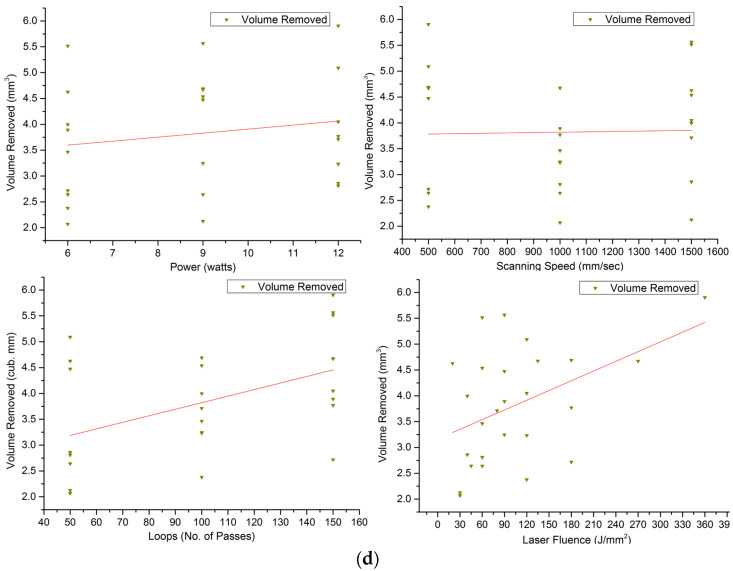
(**a**). Influence of Power (P), Scanning Speed (S), Loops (L), and Fluence (F) on Average Roughness (Ra). (**b**). Influence of Power (P), Scanning Speed (S), Loops (L), and Fluence (F) on Average Depth (D). (**c**). Influence of Power (P), Scanning Speed (S), Loops (L), and Fluence (F) on Periphery/Geometry (G). (**d**). Influence of Power (P), Scanning Speed (S), Loops (L), and Fluence (F) on Volume Removed (MR or V).

Table 5 summarises the results of the laser operation for the zirconia based on the energy fluence of the laser in terms of the average roughness (Ra), average depth of the cut (D), MR, geometry (G), and geometrical error involved while obtaining the periphery or geometry. It was noticeable that most of the obtained dimensions were smaller than intended. The following table is a hand-on datasheet as a starting point. Figure 3a represents the effects of power (P), scanning speed (S), and number of passes/loops (L) on the Ra. Greater laser powers made the surface rough and followed a well-established dispersion. On the other hand, speed had a stronger inverse correlation with Ra, which emphasised the fact that mild power with higher scanning speeds produced smoother surfaces. Roughness remained almost unaltered, with the number of passes having the least effect on the Ra values. Fluence (F) is the combination of P, S, L, and the interspacing between two consecutive hatched lines (10 μm). F had a positive correlation with Ra and loops, showing that the effect of power on Ra was higher compared to speed, regardless of individual trends [38,39,40,41]. Similarly, Figure 3b–d show the effects of the individual and combined effects (in terms of laser fluence) on D, G, and MR, respectively. The average depth and MR varied positively with P and L, except S. Nevertheless, the passes hugely decided the MR from the substrate. It was observed that ample focus of the laser engraved the substrate, however, as engraving progressed, the laser lost its focus and set power, F became insufficient, and the desired cut could only be achieved by increasing the number of passes [39,40]. Increments in S resulted in a better quality (less roughness) and was inversely proportional to Ra and D, weakly proportional to G, and constant with MR. Higher S values meant less time of laser interaction with the substrate surface and, thus, less D. Furthermore, MR was mostly driven by L after the initial engraving.

This study is important for observing the range of S along with P and L to attain the maximum production rate by keeping that engraving time at a minimum. It is evident that raising the number of passes for optimized power–speed permutation will achieve the maximum productivity by keeping the geometry within the given limits.

#### 3.1.1. Quantitative Comparison of the Influence of the Input Variables on Output Variables

Table 6 separately records nine experiments for analysing the P, S, and L individually by keeping the other two constant at a time. It was evident that, with an increase in P (6, 9, 12 watts), this resulted in an increase in roughness (67.472, 89, 138.74 μm). This happened because more energy impeded the surface at higher powers and removed bulk material, and large bulk removal from the surface caused high irregularities in the grooves. This bulk removal also caused more geometry differences, and in addition, peripheral error (G) also rose with power inputs. A very high roughness corresponding to the maximum power restricted us to finding out the MR due to limitations of the focus of the 3D optical profilometry.

Higher scanning speeds (500, 1000, and 1500 mm/s) are always favourable for a better surface quality (131.1, 83.292, and 63.962 μm), because the laser stays less at one location and melting–remelting occurs quickly. There was a substantial spike in the D and MR achieved with mounting speeds. A minute drop and rise were noted errors in geometry during the engraving [40].

As discussed earlier, increments in L had a substantially positive effect on D and MR. The geometry also changed a lot with the number of passes, because more loops passed from the periphery and removed a higher amount. It was also observed that MR increased quickly to some extent with L, however, this increment dropped with a further rise in the number of L. For experiment 25, the value of roughness was an outlier (925.8*, i.e., a through cut or a pinpoint hole where roughness shows an extremely high value) and could be a specific location on the surface with very deep penetration. A greater number of passes also means more energy, which results in a higher Ra or impaired surface quality due to frequent melting–remelting [41].

#### 3.1.2. Visual Inspection

**Figure 4:**
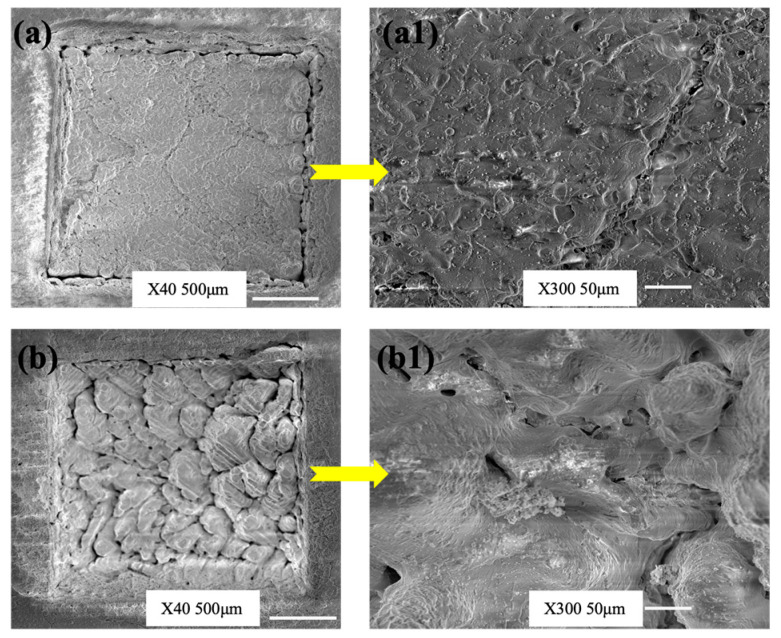
Scanning Electron Microscopy (SEM) images of green zirconia engraved by Nd: YV04 Laser (**a**) Experiment 10: P = 6 watts, S = 1500 mm/s, L = 50 and Fluence (F) = 20 J/mm^2^, (**a1**) magnified version of (**a**), (**b**) Experiment 24: P = 12 watts, S = 500 mm/s, L = 150 and Fluence (F) = 360 J/mm^2^, (**b1**) magnified version of (**b**).

A visual analysis through the Scanning Electron Microscopy (SEM, JSM-6010 LV, JEOL, Mitaka, Tokyo, Japan) of two experiments (Run Order 10 and 24) is shown in Figure 4. These cases were chosen because of the minimum and maximum laser energy fluence. Figure 4a,b show cavities, and magnified views of the bottom surfaces’ grooves are shown in the respective Figure 4a1,b1. The images clearly show that there was sintering for both cases. For experiment number 10, P and L were minimum and S was maximum, resulting in the lowest F, which gave a better surface finish, was smoother, and had cleaner cuts, however, it produced smaller geometry than intended, lesser depth, and less MR compared to experiment number 24 with P and L at the maximum and S at the minimum, resulting in the maximum F.

These values of fluence with lower power settings and a smaller number of passes were used produce better-quality cuts qualitatively and quantitatively, as mentioned earlier. However, the same inputs resulted in lower dimensions than desired and low material removal. The mechanism behind this phenomenon is that medium or low power settings caused less melting at specific location, a lower number of passes meant the number of irradiations travelling from one location were less, and higher speeds offered a small timespan at one location. Contrary to that, a maximum power (that melted a large quantity), larger number of passes (the times of irradiations passing through locations were more), and lesser speeds (slowness of the laser beam during engraving) produced rough and oversized cavities.

It was evident that any laser parametric setting would sinter the green zirconia and periphery of the cut. The only difference here was that the sintered lobes and racks generated were far bigger for the higher fluence settings, because with minimum speeds, the molten materials started cooling down and created large granules. On the other hand, with higher speeds, the material kept on melting frequently and did not solidify quickly in a smaller and finer grain size [42].

To remove the blocks of green zirconia from the palette with a better roughness, a surface study of Figure 4 is very crucial. It not only gives a qualitative picture of the MR, but also dimensional accuracy. To obtain the dimensional accuracy (especially in implants and prosthetics) of a removed piece, a balance must be struck between the parameters and productivity.

### 3.2. Finding for Stainless Steel

**Figure 5:**
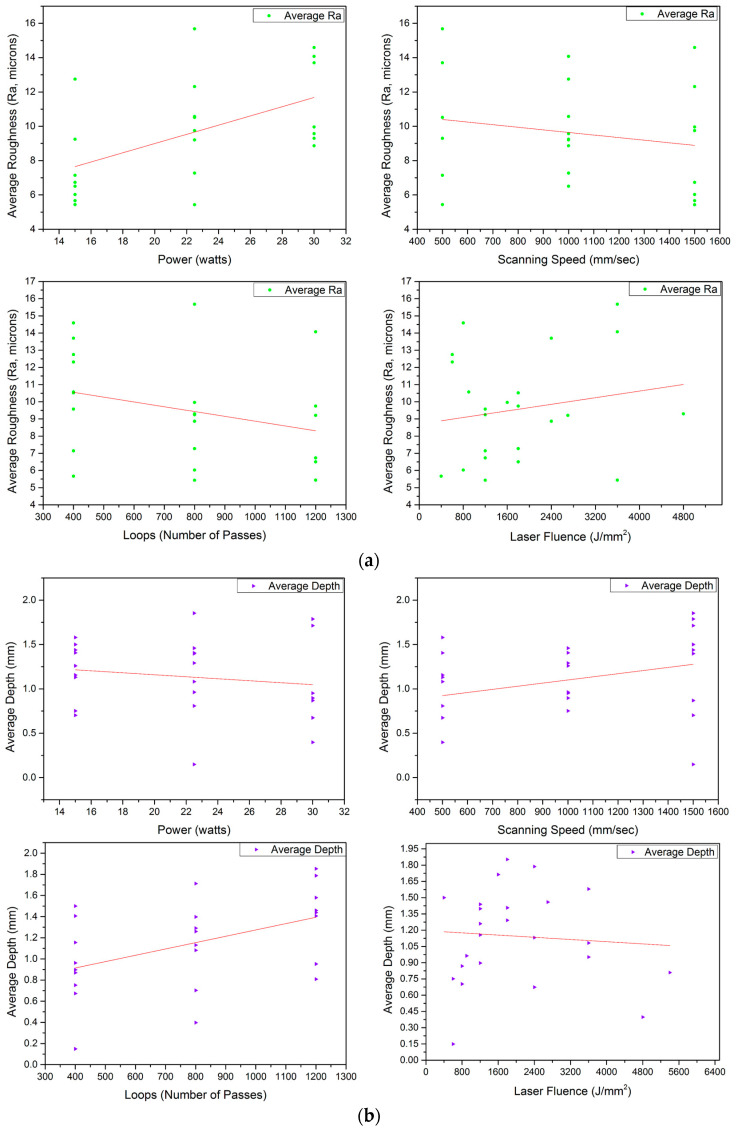

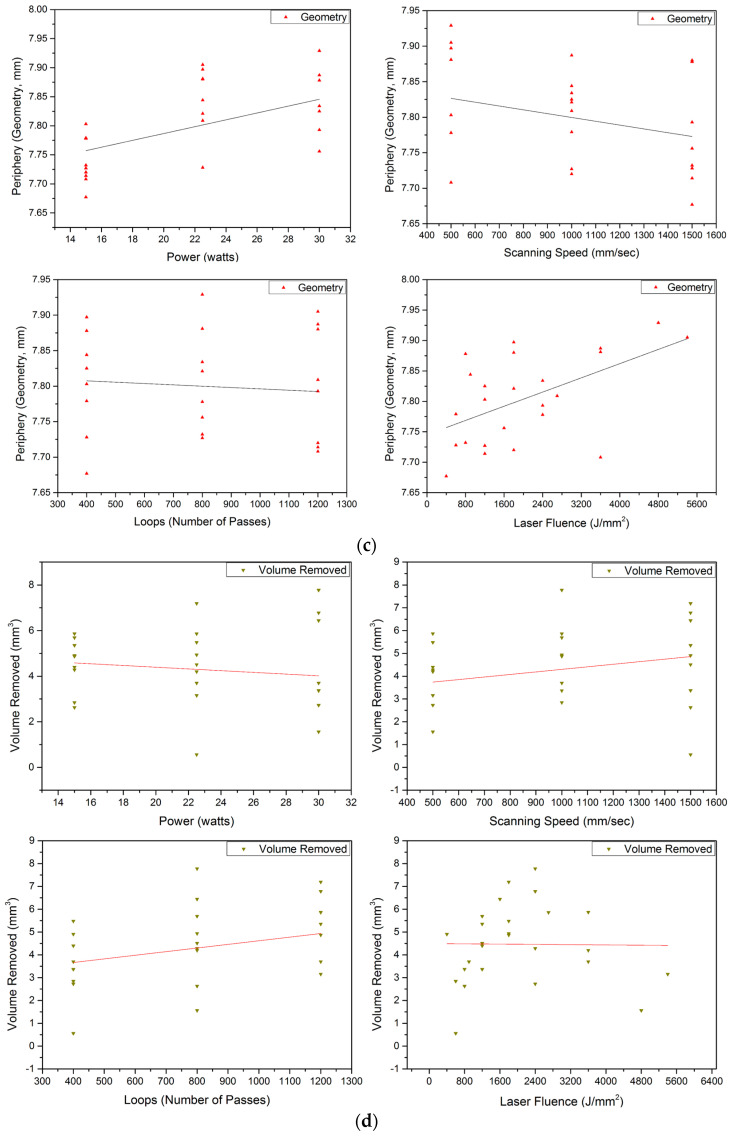
(**a**). Influence of Power (P), Scanning Speed (S), Loops (L), and Fluence (F) on Average Surface Roughness (Ra). (**b**). Influence of Power (P), Scanning Speed (S), Loops (L), and Fluence (F) on Average Depth (D). (**c**). Influence of Power (P), Scanning Speed (S), Loops (L), and Fluence (F) on Periphery/Geometry (G). (**d**). Influence of Power (P), Scanning Speed (S), Loops (L), and Fluence (F) on Volume Removed (MR or V).

Table 7 summarises the results for the SS-304 for input variables P, S, and L with respect to the outputs Ra, D, MR, G, and error in geometry from the intended dimension. It can be observed from the data that all the dimensions achieved were less (although very close to the intended values) than the intended peripheral dimension of 8 mm, unlike the zirconia, where some grooves were larger than the intended size. That signifies the fact that SS-304 must be cut to larger dimensions than those intended with given errors, and zirconia blocks will sit comfortably inside the cavity. Figure 5a–d exhibit the trends of the output values obtained by applying a set of input parameters. Figure 5a represents the increments in the average roughness with power, which followed a positive correlation, but with roughness decreasing with scanning speeds. On the other hand, a slight inverse trend was seen with L, like the zirconia engraving process. Nevertheless, the decrements in Ra with L were more prevalent in the steel. The reason for this could be the higher sensitivity of the SS-304 with temperature, unlike zirconia, which has a far greater sintering temperature. Hence, increasing L remelted the groove material and could smoothen it.

Any value less than 5 μmand above 20 μmwas not considered to maintain the consistency of the results. Although the maximum roughness quotient was almost same for the 22.5 watts and 30 watts power set ups, the uniformity observed was persistent in the 30 watts setting compared to that of 22.5 watts. This can be observed by the readings from experiments number 11 (at 22.5 watts) and 27 (at 30 watts) being 17.98 μm, 17.55 μm, 11.5 μm, 14.99 μm, 13.49 μm, and 13.50 μm, respectively. Highly non-uniform values of Ra indicated the presence of ditches at a small span of cut, giving a spike in the roughness [43,44,45,46].

Fluence (F) represents the energy concentration and interaction of the laser beam with the substrate. The more energy absorbed by the metal, the more irregularities on the surface. Sometimes, due to a material’s anisotropy, this energy interaction varies from location to location on the same material, exhibiting different levels of roughness profile. Roughness had the similar trend for SS-304 as that of zirconia with fluence. The roughness profiles can be deep pitting or pin hole types [47].

An observation of Figure 5b shows that, with a rise in power from 15 to 22.5 watts, D increased but started decreasing by further increasing the P. That implies that an increase in power will not necessarily increase the depth of the cut. Moreover, at a low P like 15 watts, the D values obtained were coherent and dispersed in a narrow vertical range, unlike with the power settings of 22.5 or 30 watts. Considering S against D showed an overall positive trend. There is a very crucial observation presenting a rise in D at a higher S, i.e., 1500 mm/s though D suffered when the S increased from 500 to 1000 mm/s for some combinations. Like zirconia, SS-304 also showed a strong positive correlation obtained for D and L. D followed downward trend for a rise in F. This phenomenon can be attributed to the energy dissipation effect of metal, unlike zirconia. SS-304 will dissipate the heat faster and higher S values will not decrease the energy concentration, resulting in an overall loss in D values. The dominance of P and L was visible and consistent (similar to zirconia) from the run order 24, i.e., a cut penetrating the plate due to the highest fluence energy of 7200 J/mm^2^ for the minimum speed [46]. Based on the analyses of zirconia, the variations in F can be drawn for SS-304 as well.

The geometry of SS-304 grooves is crucial, because the ceramic blocks must fit inside them tightly, creating a strong adhesion bond with the adhesive glue interface. Periphery or geometrical dimension were also used to determine the volume of the removed material. Greater P settings positively influenced the G by clearing out the periphery, as shown in Figure 5c, however, S showed a strong negative trend. This occurred due to the lower concentration of the laser beam on a location at higher speeds. The effect of P dropped while moving inside the cut, because of the absorption and reflection of the laser beam and interaction with the oxidized metal [48]. L hardly influenced the G, as the laser moving inside the cavity converged and depended mostly on the power of the laser due to beam focus after the initial cuts. This phenomenon can be observed by analysing L changing from 400 to 800 to 1200, slightly increasing the circumference but then almost remaining the same for all loop values. Besides the weak effect of L, F showed positive correlation due to the heavyweight P in the equation.

As discussed earlier for zirconia, P alone cannot dictate the MR values. It is visible from Figure 5d that P in relation to MR had a horizontal to negative tendency. With the scanning speeds and L, MR increased, but F showed a horizontal trend, i.e., the increases in P and L were balanced by a rise in S. Table 7 presents that large F values removed bulk material, and hence, overall parametric combination and optimisation were necessary, rather than independently changing the variables [48,49,50].

#### 3.2.1. Qualitative Inspection

**Figure 6:**
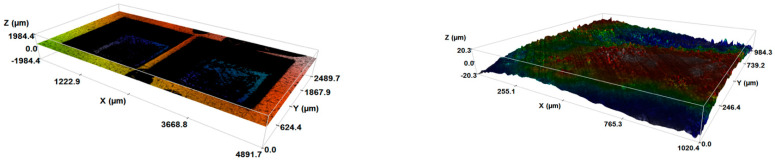
Engraved grooves of Experiment 4 (15 W, 500 mm/s and 400 loops) at (**left**); roughness of the bottom surface for Experiment 4 at (**right**).

The quality and morphology of the cut can be seen in Figure 6. Figure 6 shows a depiction of a perfectly removed engraved cavity and its corresponding bottom surface roughness. The engraved quality was excellent, as achieved by a cross-hatched filled design, and the edges obtained were perfectly square with very limited curvature. Thus, the study will be found helpful in qualitative and quantitative ways, and other materials can also be explored with other metals and ceramics based on this study for aesthetic and design purposes [35].

#### 3.2.2. Visual Inspection

**Figure 7:**
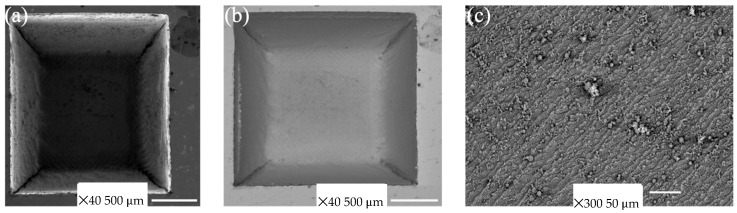
Scanning Electron Microscopy (SEM) images of laser-engraved SS-304 (Experiment-11); (**a**) cut groove, (**b**) cut profile, and (**c**) roughness of the bottom.

**Figure 8:**
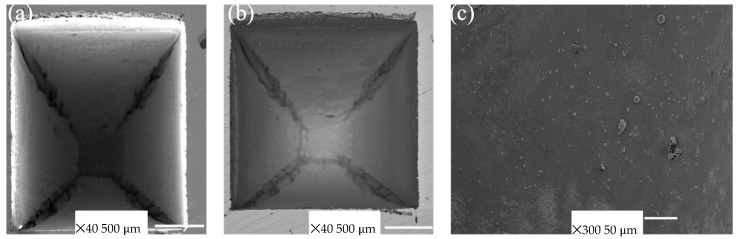
Scanning Electron Microscopy (SEM) images of laser-engraved SS-304 (Experiment-17); (**a**) cut groove, (**b**) cut profile, and (**c**) roughness of the bottom.

From Figure 7 and Figure 8, it can be visualized that this laser engraving offered very efficient material removal without leaving much debris or HAZ because of the compressed inert argon environment. For a visual analysis, the chosen experiments were 11 (F = 400 J/mm^2^) and number 17 (Fluence = 5400 J/mm^2^), with intact bottom surfaces. It is not desired in the case of cutlery engraving to oxidise the surface, as aesthetics are the prime requirement, hence, an optimised combination of P, S, and L can only prove to be proper.

Figure 7a–c and Figure 8a–c represent the SEM images of the engraved cut for the minimum F values. With a high F, the groove became narrower with depth due to the wall reflection and defocusing of the laser beam (Figure 8a). On the other hand, with less F, the cuts were not very deep, and the convergence of the laser beam was low as well (Figure 7a), resulting in a less conical groove. A lower F could mean lower values of P or L or higher values of S, which would result in a better surface finish, clean grooves, less depth of the cut, and material removal (a higher S provided most of these outcomes, so it is appropriate to say that this low F was a result of high S values). A high F could increase D with a large L (Experiment 11 has 400 and 17 has 1200 passes), but as the conicity increased, MR decreased gradually, resulting in less MR. Therefore, a higher F did not guarantee a greater MR.

## 4. Conclusions

The study presents a thorough picture of the correlation of input laser parameters with output variables and their optimisation. The obtained outcomes were as follows:

(i) The total energy of the laser or fluence offered the full scenario of the surface quality (roughness), material removal (volume removed and depth of cut), and cut dimensions (geometry or periphery).
(ii) Material removal with a high surface quality and clean-cut cavity defined the productivity. A combination of high-quality engraving with the least possible time of production was favourable.
(iii) It was found that fluence had positive correlation with all the output variables, regardless of the individual trends of the input power, scanning speed, or number of passes.
(iv) The constant positive trend of fluence with respect to the outputs was mostly due to the drop or constancy of the scanning speeds against the roughness (better finish), depth of cut, geometry, or volume removed, rather than the dominance of power or number of passes.
(v) The study proved that ceramics like zirconia and metals like stainless steel do not behave extremely different from each other when it comes to laser interactions. However, the parametric optimisation is entirely distinct for both.
(vi) More power values attracted more roughness with constant geometry by maintaining depth, volume removed, and constant geometry, except geometry for steel, where the dimension increased with the power settings.
(vii) Higher scanning speeds provided a better surface finish, without any exceptions for both substances.
(viii) A higher number of passages/passes meant more material removed for both materials.
(ix) Medium power settings and higher scanning speeds with the maximum number of passes produced the best outcomes.

We segregated zirconia blocks from zirconia palettes and engraved SS-304 with these optimised parameters, producing a wide variety of patterns on cutlery as graphical abstract exhibits. The integration of perfectly safe, wear-resistant, antimicrobial, colourful, and aesthetically appealing zirconia on cutlery is an attractive perspective. Moreover, zirconia does not corrode, oxidise, or change its composition with time. Hence, it is safe to say that the process of laser engraving is a proven, accurate, fast, and affordable way to produce state-of-the-art flatware/houseware/cutlery without making huge changes in the overall production process.

## Figures and Tables

**Table 1:** Specifications of zirconia powder used for palettes.

ZrO_2_ (%wt)	Al_2_O_3_ (%wt)	Y_2_O_3_ (%wt)	HfO_2_ (%wt)	MgO	NaO_2_	SiO_2_, K_2_O, CaO, Fe_2_O_3_
73.1	20	4	<2	200	<40	<30
**Average Crystallite Size**	**Minimum Purity** **(Zr + Y + Hf + Al)**	**Alumina** **Content**	**Specific Surface Area**	**Granulate Size**		**Intercept Zr/Al Grain Size**
Al:150/Zr:50 nm	99.9%	20%	15 ± 2 m^2^/gm	35 μm		0.4/0.6 μm

**Table 2:** Specification of SS-304 used for laser engraving.

Element	C	Mn	Si	Cr	Ni	N
Weight (%)	≤0.08	≤2.00	0.75	18.00–20.00	8.00–10.50	0.10

**Table 3:** Full factorial DOE of zirconia for 3 variables at 3 levels for random runs.

Standard Order	Run Order	Power (watts)	Speed (mm/s)	Loops
14	1	9	1000	100
15	2	9	1000	150
13	3	9	1000	50
1	4	6	500	50
18	5	9	1500	150
23	6	12	1000	100
3	7	6	500	150
6	8	5	1000	150
17	9	9	1500	100
7	10	6	1500	50
25	11	12	1500	50
2	12	6	500	100
4	13	6	1000	50
24	14	12	1000	150
27	15	12	1500	150
9	16	6	1500	150
9	16	6	1500	150
22	17	12	1000	50
19	18	12	500	50
8	19	6	1500	100
5	20	6	1000	100
10	22	9	500	50
20	23	12	500	100
21	24	12	500	150
16	25	9	1500	50
11	26	9	500	100
12	27	9	500	150

**Table 4:** Full factorial DOE of SS-304 for 3 variables at 3 levels for random runs.

Standard Order	Run Order	Power (watts)	Speed (mm/s)	Loops
17	1	22.5	1500	800
6	2	15	1000	1200
8	3	15	1500	800
1	4	15	500	400
24	5	30	1000	1200
10	6	22.5	500	400
20	7	30	500	800
14	8	22.5	1000	800
16	9	22.5	1500	400
11	10	22.5	500	800
7	11	15	1500	400
26	12	30	1500	800
3	13	15	500	1200
15	14	22.5	1000	1200
19	15	30	500	400
4	16	15	1000	400
12	17	22.5	500	1200
18	18	22.5	1500	1200
22	19	30	1000	400
13	20	22.5	1000	400
5	21	15	1000	800
27	22	30	1500	1200
9	23	15	1500	1200
21	24	30	500	1200
2	25	15	500	800
23	26	30	1000	800
25	27	30	1500	400

**Table 5:** Results obtained for zirconia from the laser operation in terms of average surface roughness, average depth, volume removed, geometry, and error in geometry.

Experiment Number/Run Order	Fluence (J/mm^2^)	Average Ra Value (μm)	Average Depth (mm)	Volume Removed (mm^3^)	Periphery/Geometry (mm)	Designed Periphery (mm)	Error in Geometry (mm)
1	90	112.38	0.8613	3.244735691	7.767	8	−0.233
2	135	89.151	1.0893	4.673096753	8.289	8	0.289
3	45	112.95	0.6353	2.641554625	8.165	8	0.165
4	60	155.69	0.6753	2.641637919	7.909	8	−0.091
5	90	89.41	1.5253	5.565035285	7.642	8	−0.358
6	120	171.44	0.914	3.232497186	7.526	8	−0.474
7	180	125.09	0.7153	2.716376041	7.796	8	−0.204
8	90	105.59	0.977	3.892468631	7.985	8	−0.015
9	60	74.337	1.1503	4.536273931	7.943	8	−0.057
10	20	56.702	1.1757	4.62732642	7.936	8	−0.064
11	40	97.351	0.743	2.859873127	7.852	8	−0.148
12	120	131.1	0.6173	2.378056587	7.849	8	−0.151
13	30	112.36	0.609	2.06845023	7.374	8	−0.626
14	180	125.9	1.063	3.76887341	7.534	8	−0.466
15	120	138.74	1.209	4.049890065	7.324	8	−0.676
16	60	67.472	1.486	5.513271012	7.705	8	−0.295
17	60	162.97	0.6487	2.810343468	8.334	8	0.334
18	120	224.63	1.2913	5.091225005	7.944	8	−0.056
19	40	63.962	1.0373	3.99795528	7.853	8	−0.147
20	60	83.292	0.888	3.463372716	7.899	8	−0.101
21	80	124.7	1	3.713076	7.708	8	−0.292
22	90	160.43	1.1907	4.473421585	7.754	8	−0.246
23	240	38.214	0.1543	0.573599912	7.712	8	−0.288
24	360	141.88	1.3673	5.905205017	8.313	8	0.313
25	30	925.88	0.5413	2.125011037	7.927	8	−0.073
26	180	306	1.152	4.68908928	8.07	8	0.07
27	270	214.56	1.112	4.670163144	8.198	8	0.198

**Table 6:** Comparison of the influence of the input parameters on output variables.

Basis for Comparison	Experiment Number	Ra (μm)	D (mm)	V (mm^3^)	G-Error Values
Power (P)	16 5 15	67.472 89.41 138.74	1.486 1.5253 1.209	5.513271012 5.565035285 4.049890065	−0.295 −0.358 −0.676
Scanning Speed (S)	12 20 19	131.1 83.292 63.962	0.6173 0.888 1.0373	2.378056587 3.463372716 3.99795528	−0.151 −0.101 −0.147
Loops (L)	25 9 5	925.8* 74.337 89.41	0.5413 1.1503 1.5253	2.125011037 4.536273931 5.565035285	−0.073 −0.057 −0.358

**Table 7:** Results obtained from the laser operation in terms of average surface roughness, average depth, volume removed, geometry, and error in geometry.

Experiment Number/Run Order	Fluence (J/mm^2^)	Ra Value (µm)	Average Depth (mm)	Volume Removed (mm^3^)	Periphery (mm)	Designed Periphery (mm)	Error in Geometry (mm)
1	1200	5.42633	1.39745	4.25614	6.63	8	−1.37
2	1800	6.5	1.40666	4.87007	7.72	8	−0.28
3	800	6.02525	0.70233	2.62383	7.732	8	−0.268
4	1200	7.14111	1.15566	4.39583	7.803	8	−0.197
5	3600	14.07	0.952	3.69948	7.887	8	−0.113
6	1800	10.518	1.40566	5.47762	7.897	8	−0.103
7	4800	8.91777	0.39733	1.56083	7.929	8	−0.071
8	1800	7.64388	1.291	4.93584	7.821	8	−0.179
9	600	12.5577	0.149	0.55596	7.728	8	−0.272
10	3600	14.9088	1.082	4.20002	7.881	8	−0.119
11	400	5.565	1.5	5.52423	7.677	8	−0.323
12	1600	9.49155	1.713	6.43871	7.756	8	−0.244
13	3600	5.43266	1.57933	5.86372	7.708	8	−0.292
14	2700	8.99677	1.459	5.56027	7.809	8	−0.191
15	2400	14.2233	0.67333	2.72222	7.990	8	−0.001
16	600	13.1355	0.75133	2.84050	7.779	8	−0.221
17	5400	148.56 *	0.80833	3.15538	7.905	8	−0.095
18	1800	9.75	1.85266	7.18772	7.88	8	−0.12
19	1200	10.1795	0.89666	3.43102	7.825	8	−0.175
20	900	11.1255	0.963	3.70286	7.844	8	−0.156
21	1200	9.41744	1.25966	5.68803	7.727	8	−0.273
22	2400	3.1086 *	1.787	6.78121	7.793	8	−0.207
23	1200	6.62722	1.43866	5.35040	7.714	8	−0.286
24	7200	Through *	Through *	Through *	7.687	8	−0.313
25	2400	4.2696 *	1.13133	4.27768	7.778	8	−0.222
26	2400	9.07511	2.02733	7.77585	7.834	8	−0.166
27	800	14.59	0.869	3.37069	7.878	8	−0.122

* The outliers i.e., the values that are extremely high or extremely low.

## Data Availability

Data are contained within the article.
